# Semantic Segmentation of Satellite Images for Landslide Detection Using Foreground-Aware and Multi-Scale Convolutional Attention Mechanism

**DOI:** 10.3390/s24206539

**Published:** 2024-10-10

**Authors:** Chih-Chang Yu, Yuan-Di Chen, Hsu-Yung Cheng, Chi-Lun Jiang

**Affiliations:** 1 Department of Information and Computer Engineering, Chung Yuan Christian University, Taoyuan City 320, Taiwan; ccyu@cycu.edu.tw; 2 Department of Computer Science and Information Engineering, National Central University, Taoyuan City 320, Taiwan

**Keywords:** remote sensing, semantic segmentation, convolutional attention mechanism, multi-scale features fusion

## Abstract

Advancements in satellite and aerial imagery technology have made it easier to obtain high-resolution remote sensing images, leading to widespread research and applications in various fields. Remote sensing image semantic segmentation is a crucial task that provides semantic and localization information for target objects. In addition to the large-scale variation issues common in most semantic segmentation datasets, aerial images present unique challenges, including high background complexity and imbalanced foreground–background ratios. However, general semantic segmentation methods primarily address scale variations in natural scenes and often neglect the specific challenges in remote sensing images, such as inadequate foreground modeling. In this paper, we present a foreground-aware remote sensing semantic segmentation model. The model introduces a multi-scale convolutional attention mechanism and utilizes a feature pyramid network architecture to extract multi-scale features, addressing the multi-scale problem. Additionally, we introduce a Foreground–Scene Relation Module to mitigate false alarms. The model enhances the foreground features by modeling the relationship between the foreground and the scene. In the loss function, a Soft Focal Loss is employed to focus on foreground samples during training, alleviating the foreground–background imbalance issue. Experimental results indicate that our proposed method outperforms current state-of-the-art general semantic segmentation methods and transformer-based methods on the LS dataset benchmark.

## 1. Introduction

Taiwan is situated at the intersection of the Philippine Sea Plate and the Eurasian Continental Plate, leading to extremely steep mountainous terrain with fragile rock structures. The frequency of earthquakes, coupled with concentrated rainfall from typhoons and the early-summer rainy season, exacerbates the risk of landslides. These factors make Taiwan one of the most landslide-prone regions in the world. To effectively respond to landslide disasters, precise detection of affected areas and damage assessment are essential.

**Figure 1:**
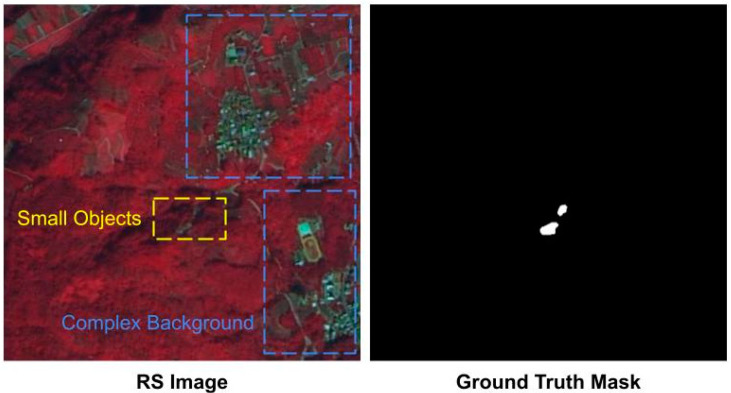
The challenges of high-resolution remote sensing imagery.

Landslides are typically detected using high spatial resolution (HSR) remote sensing imagery. Through semantic segmentation, the goal is to accurately identify and extract landslide areas from these images. HSR satellite imagery provides precise semantic and locational information, making it widely used in many remote sensing applications, such as urban planning, monitoring, and disaster assessment [1,2,3,4]. However, semantic segmentation in HSR remote sensing imagery is more challenging than in natural scenes, mainly due to the following reasons:

1. Multi-Scale Problem [5,6]: In HSR remote sensing images, objects often have larger scale variations. This complicates localization and identification, as models must handle a range of object sizes from small to large. In [7], the authors propose a multiresolution fusion module to extract and integrate features from coarse to fine details, and a selective kernel attention module to optimize the fused multiresolution information using progressively increasing receptive fields. In [8], a shape-enhanced vision transformer model is proposed to extract and keep the multi-scale shape information of landslides.
2. Complex Backgrounds [9]: HSR remote sensing images typically have more complex backgrounds, leading to greater intra-class variation. This makes the model harder to distinguish between the foreground and background, increasing the likelihood of false alarms. Figure 1 shows that the blue box contains numerous houses, land, and other features, all regarded as background but with a huge appearance variation.
3. Foreground–Background Imbalance [10]: Remote sensing images have a much lower proportion of foreground objects than natural images. This results in models excessively incorporating background information, neglecting numerous small foreground targets (as shown by the yellow box in Figure 1, which is a small landslide area).

Large-scale variation problems are common in most semantic segmentation tasks. In natural scenes, current semantic segmentation is usually limited by the multi-scale problem, so general methods often focus on multi-scale context modeling [11,12,13,14]. However, in HSR remote sensing imagery, these general methods seldom address the issues of false alarms and the foreground–background imbalance. The lack of explicit foreground modeling hinders the advancement of semantic segmentation in remote sensing images.

To address the aforementioned issues, this study introduces a multi-scale convolutional attention mechanism [15] in the encoder and adopted a Feature Pyramid Network (FPN) [14] architecture to extract multi-scale features, thereby mitigating the multi-scale problem. To suppress false alarms, the model incorporates a foreground–scene relationship module to model the foreground and scene to enhance foreground features. To tackle the foreground–background imbalance, a Soft Focal Loss [16] is used in the loss function, focusing on foreground samples during training.

## 2. Related Works

Semantic segmentation is a fundamental task in computer vision. Inspired by Fully Convolutional Networks [17] (FCNs), semantic segmentation replaces the fully connected layer with a convolutional layer. This approach allows for an input of images of any size to achieve pixel-level predictions. It is the first pixel-to-pixel semantic segmentation method and is also end-to-end trainable. DeepLab v1 [18] utilizes atrous convolution to expand the receptive field of the Convolutional neural network (CNN), exploiting spatial context for semantic segmentation. Atrous Spatial Pyramid Pooling [19] (ASPP) and the Pyramid Pooling Module [13] (PPM) use multiple atrous convolutions with different atrous rates and pyramid pooling [20] to achieve multi-scale feature extraction. The “encoder-decoder” network architecture [21,22] restores the spatial resolution of features. A self-attention mechanism [23] has been introduced into visual tasks to capture richer contextual dependencies.

### 2.1. Attention Mechanisms

Attention mechanisms are adaptive selection processes designed to guide the network’s focus towards important information. The Visual Attention Network [24] (VAN) and SegNeXt [15] use Multilayer Perceptron (MLP) and large-kernel convolutions to extract global features instead of the self-attention mechanism employed by transformers. SegNeXt designs parallel convolutions with different kernel sizes to extract multi-scale features and uses a 1 × 1 convolution to achieve channel attention [25], which is ignored by the self-attention mechanism.

### 2.2. Multi-Scale Feature

Designing multi-scale networks is a prominent trend in computer vision. GoogleNet [26] employs a multi-branch structure for extracting multi-scale features. HRNet [27] employs parallel branches at multiple resolutions, integrating high-resolution features with low-resolution features to achieve multi-scale feature fusion while preserving high-resolution features in deeper stages. FPN [14] is a commonly used architecture for addressing multi-scale challenges. It combines the concepts of top-down and lateral connections, ensuring each layer has sufficient high-level semantics to detect small objects and retain positional information lost during up-sampling and down-sampling processes.

### 2.3. Semantic Segmentation of HSR Images

Semantic segmentation techniques find extensive application in remote sensing, spanning various areas such as road extraction, vehicle detection, land cover classification, urban planning, and disaster detection. The relation network [28] captures distant spatial relationships between entities through spatial and channel relation modules. FarSeg [9] introduces a foreground-aware relation network to model the relationship between foreground objects and the geospatial scene using a 1-D scene embedding vector.

## 3. Purposed Methods

### 3.1. Model Architecture

**Figure 2:**
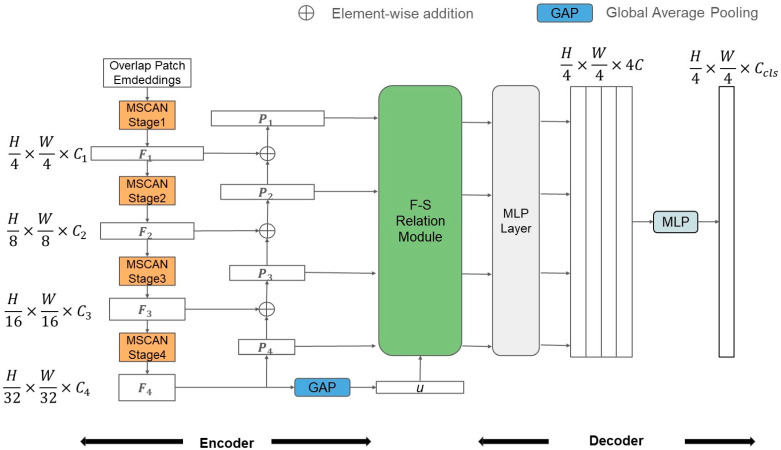
Overall architecture of the proposed system.

As shown in Figure 2, the proposed model is primarily divided into two parts: the encoder and the decoder. The encoder employs an attention mechanism designed with multiple convolution layers of different sizes. Inspired by the FPN [14] concept, the encoder fuses the feature maps output from different layers and connects them laterally. This approach not only extracts multi-scale features but also supplements the feature information lost during the down-sampling process. Before the feature maps are fed into the decoder, they pass through the Foreground–Scene Relation Module [9] (F–S Relation Module). This module is designed to learn the relationship between the foreground and the scene. The decoder consists solely of a Multilayer Perceptron (MLP), which directly integrates the feature maps for prediction.

Initially, we divide the input image into multiple overlapping patches. The encoder’s backbone network uses the SegNeXt [15], combining convolution and attention mechanisms. It employs multiple large-kernel convolution branches to design multi-scale convolutional attention, with $F_{i}$ representing the feature map output from each layer of the multi-scale attention network. A pyramid structure is utilized, where $P_{i}$ denotes the feature map after the pyramid structure. $P_{4}$ is obtained from $F_{4}$ via a 1 × 1 convolution. From $F_{3}$ to $F_{1}$, each $F_{i}$ is fused with $P_{i+1}$. In addition to the pyramid, $F_{4}$ undergoes global average pooling (GAP) to produce an embedding vector u. The pyramid outputs, $P_{i}$ and u, are fed into the F–S Relation Module, where u is element-wise multiplied with each pyramid layer output to obtain new feature maps $Z_{i}$.

Finally, the lightweight decoder using only MLPs is employed. After aligning dimensions and performing up-sampling, the feature maps from each layer are concatenated. The final MLP produces a set of channels equal to the number of classes, which performs the final prediction. The term $C_{cls}$ is denoted as the channel number of classes.

### 3.2. Encoder

**Figure 3:**
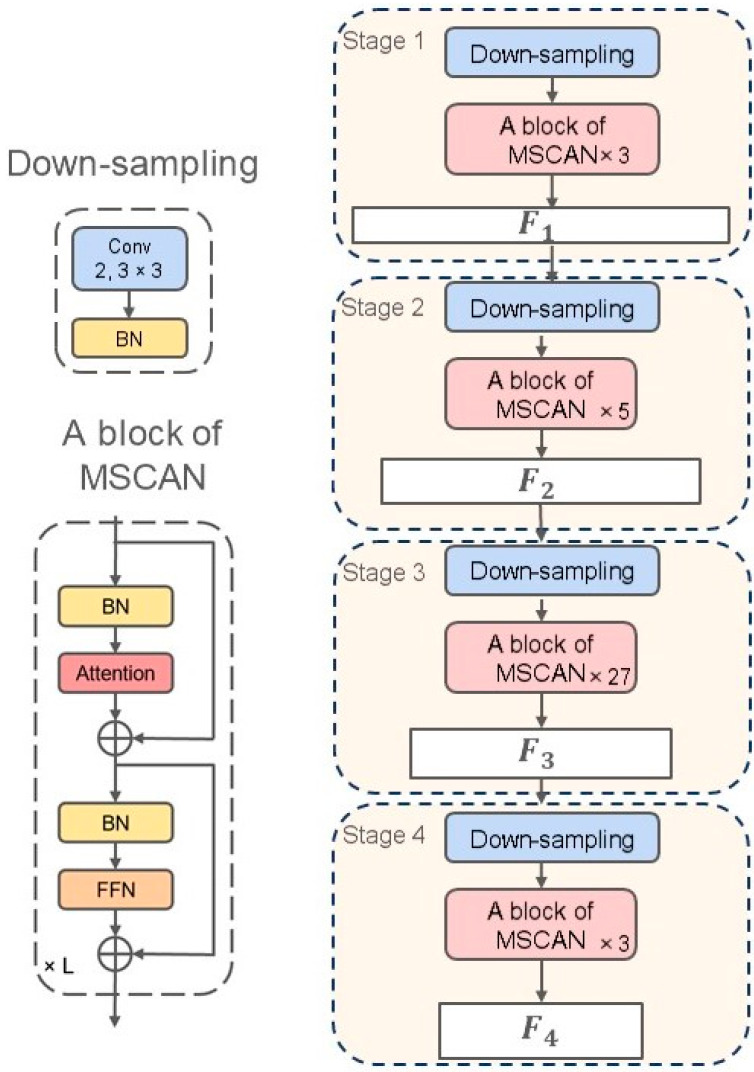
Backbone architecture using MSCAN.

The backbone network employs the Multi-Scale Convolutional Attention Network (MSCAN) by SegNeXt [15] and is divided into four stages. As shown in Figure 3, each stage consists of a down-sampling block and a stack of multiple MSCAN blocks, producing four feature maps with progressively reduced spatial resolutions. The feature map of each stage is denoted as $F_{i}$. The detailed sizes and channel numbers of the four feature maps are shown in Table 1, where *H* and *W* represent the image height and width, and $C_{i}$ represents the number of each channel. As shown in Table 1, the spatial resolution gradually decreases by a factor of two at each stage. As the resolution decreases, the number of output channels increases, with *L* indicating the number of MSCAN blocks required for each stage. After down-sampling, the output size remains constant for all subsequent layers within the stage. Within these layers, *L* sets of MSCAN blocks are stacked.

#### 3.2.1. Multi-Scale Convolutional Attention Network (MSCAN)

**Figure 4:**
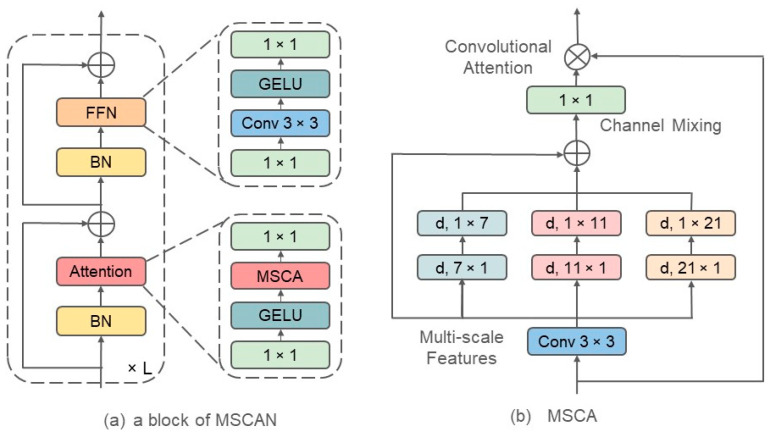
A block of Multi-Scale Convolutional Attention Network.

The MSCAN block adopts a structure similar to ViT [29], as shown in Figure 4a, and includes two residual blocks: an attention mechanism and a Feed-Forward Network (FFN). Unlike ViT, the MSCAN replaces self-attention with Multi-Scale Convolutional Attention (MSCA). Initially, the input is through Batch Normalization (BN) and then enters the attention mechanism composed of MSCA. The output from this mechanism is added to the original input, forming a residual connection. This is followed by another BN layer and an FFN, concluding with an additional residual connection. The attention mechanism consists of a 1 × 1 convolution, GELU activation, MSCA, and another 1 × 1 convolution. The FFN comprises a 1 × 1 convolution, a 3 × 3 depth-wise convolution, GELU activation, and a final 1 × 1 convolution.

To enhance multi-scale feature information, the Multi-Scale Convolutional Attention mechanism (MSCA) is introduced. In the MSCA, different sizes of convolutional kernels enable multi-scale feature extraction. The MSCA consists of three components, as illustrated in Figure 4b:

1. A depth-wise convolution for aggregating local information.
2. Multi-branch strip depth-wise convolutions to capture multi-scale information.
3. A 1 × 1 convolution for channel fusion.

The output of the 1 × 1 convolution serves as the attention weights to re-weight the input to the MSCA. Mathematically, the MSCA can be written as follows:

(1) $$\mathrm{Att}={Conv}_{1\times 1}\left(\sum_{i=0}^{3}{Scale}_{i}\left(\mathrm{DW}-\;\mathrm{Conv}\left(F\right)\right)\right)$$

(2) Out=Att × F

where F represents the input features. Att and Out denote the attention map and the output, respectively. The notation “×” indicates element-wise multiplication. DW-Conv stands for depth-wise convolution, and $\left\{{Scale}_{i},\;i\in 0,1,2,3\right\}$ represents the *i*-th branch in Figure 4b, where ${Scale}_{0}$ is the direct input.

In each branch, the kernel size is set to 7, 11, and 21. To reduce the computational burden and the number of parameters associated with large-kernel depth-wise convolutions, each large-kernel depth-wise convolution is replaced with two depth-wise separable convolutions. Specifically, a k×k convolution is decomposed into a k×1 convolution followed by a 1×k convolution. For instance, a 7×7 2-D convolution requires 7×7=49 parameters. By decomposing it into a pair of 7×1 and 1×7 convolutions, only 14 parameters are needed.

This approach not only reduces the number of parameters but also enhances the efficiency of the model by streamlining the convolution operations across multiple scales, as shown in Figure 4b. The MSCA effectively utilizes multi-scale convolutional attention to dynamically adjust the focus across different feature levels. Additionally, it minimizes overfitting and improves generalization by limiting the complexity of the architecture of the model.

#### 3.2.2. Feature Pyramid Networks (FPN)

**Figure 5:**
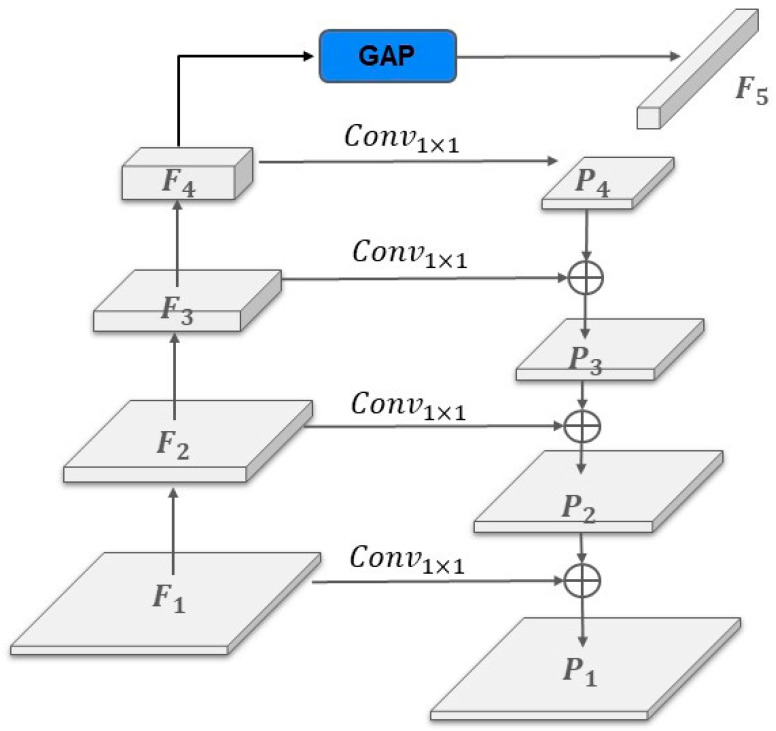
Feature Pyramid Networks.

The set of feature maps extracted from the backbone network denoted as $\left\{F_{i}\;|\;i=1,\;2,\;3,\;4\right\}$ are used to generate a pyramid of feature maps with the same number of channels $\left\{P_{i}\;|\;i=1,\;2,\;3,\;4\right\}$. As shown in Figure 5, this process employs the top-down pathway and lateral connections of FPN [14]. This process is described as Equation (3).

(3) $$P_{i}={\mathrm{Conv}}_{1\times 1}\left(F_{i}\right)+{Up}_{2\times }\left(P_{i+1}\right),\;i=4,\;3,\;2,\;1$$

Here, ${Conv}_{1\times 1}$ represents a 1 × 1 convolution used for lateral connections, and ${Up}_{2\times }$ denotes nearest neighbor up-sampling with a scaling factor of two. The combination of the top-down pathway and lateral connections helps recover information lost during down-sampling and supports multi-scale feature extraction. Additionally, $F_{4}$ undergoes global average pooling to generate a scene feature $F_{5}$, as shown in Equation (4).

(4) $$\;F_{5}=GAP(F_{4})$$

The scene feature is used to establish the relationship between foreground and scene, to be detailed in the next subsection.

The design of this structure incorporates a top-down pathway and lateral connections that fuse each layer of feature maps with the preceding one, ensuring each layer is endowed with sufficient high-level semantics to detect small objects. Without lateral connections, although the top-down pathway provides each layer with finer resolution and semantics, positional information would be lost due to the up-sampling and down-sampling processes. Furthermore, in addition to reducing computational load, the combination of top-down and lateral connections not only conserves resources but also enhances the detection of small objects by fully utilizing the feature maps generated layer-by-layer in the CNN, ensuring that each feature map in the pyramid possesses extensive semantic information. For remote sensing images where there is an imbalance between the foreground and the scene, this method effectively extracts foreground features and their positional information, mitigating the impact of the foreground features being significantly less pronounced than the scene features.

### 3.3. Foreground–Scene Relation Module

**Figure 6:**
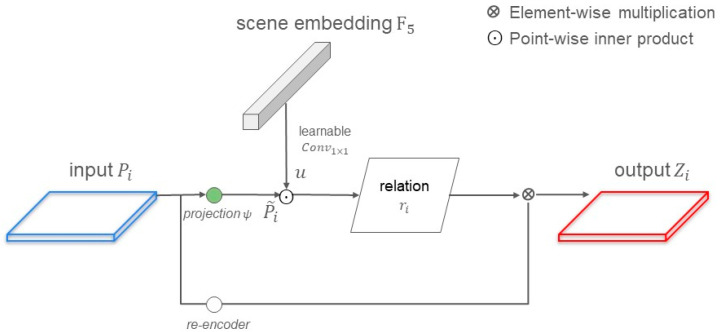
Detail of layer *i* in the foreground–scene relationship module.

The Foreground–Scene Relation Module (F–S Relation Module) is designed to enhance the discriminative ability of foreground features. In high spatial resolution (HSR) remote sensing imagery, it often has highly complex backgrounds, resulting in greater intra-class variance within the background, which can lead to misclassification issues. To address this problem, the F–S Relation Module establishes a relationship between the foreground and the scene, using this relationship to strengthen foreground features and increase the difference between foreground and background features. This approach allows for more effective differentiation between the foreground and background, thus improving the model’s accuracy. As illustrated in Figure 6, the input consists of the pyramid feature maps $P_{i}$. The F–S Relation Module generates new feature maps $Z_{i}$ by re-encoding $P_{i}$ and re-weighting them using a relation map $r_{i}$.

The relation map $r_{i}$ is a matrix that describes the similarity between the scene and the foreground. To align *u* and $P_{i}$, two projection functions are used. The transformed feature map ${\tilde{P}}_{i}$ is computed by applying the scale-aware projection function ψ to $P_{i}$, as shown in Equation (5):

(5) $${\tilde{P}}_{i}=\psi \left({\;P}_{i}\right)$$

where ψ(·) is implemented by a 1 × 1 convolution layer followed by Batch Normalization and ReLU activation function in order. The scene embedding vector *u* is calculated by applying a learnable ${Conv}_{1\times 1}$ to $F_{5}$, as shown in Equation (6).

(6) $$u={Conv}_{1\times 1}\left({\;F}_{5}\right)$$

The purpose of the 1×1 convolution and the projection function ψ(·) is to ensure that foreground feature maps $P_{i}$ and the scene embedding $F_{5}$ have the same number of channels, enabling the calculation of the relation map $r_{i}$. Since the geospatial scene semantics are consistent across different scales, the scene embedding vector *u* is shared among all pyramid feature maps. The relation map $r_{i}$ is obtained by calculating the similarity estimation function through a point-wise inner product between u and ${\tilde{P}}_{i}$, as shown in Equation (7).

(7) $${\;r}_{i\;}=u⊙{\tilde{P}}_{i}$$

In our research, the scene embedding vector *u* represents an abstract depiction of the overall scene, while each local vector in the feature map ${\tilde{P}}_{i}$ represents the characteristics of specific regions within the scene. By computing the dot product between u and ${\tilde{P}}_{i}$, we can quantify the degree of match between the scene embedding vector and each local feature, thereby generating a relation map.

Based on a simple self-gating mechanism, the Sigmoid gating function is used to normalize $r_{i}$. The relation map $r_{i}$ is utilized to weight the original feature map of the re-encoder. The enhanced relational foreground feature map $Z_{i}$ is calculated using Equation (8). Due to the nature of the dot product, when the foreground features are similar to the scene features, the corresponding $r_{i}$ values are larger; conversely, they are smaller when dissimilar. Through the design of Equation (8), we use the relation map to weight the features of the re-encoder, thereby modulating the importance of the original feature map. In this way, parts of the relation map with higher values, which correspond to features highly relevant to the scene, are enhanced, while those with lower values, representing less relevant features, have their weights diminished.

(8) $$Z_{i}=\frac{1}{1+e^{-r_{i}}}\times \mathrm{re}-\mathrm{encoder}\left(P_{i}\right)$$

Since the weighting operation is a linear function, an additional nonlinear unit is designed to prevent feature degradation. Therefore, a streamlined encoder consisting of a 1 × 1 convolution layer, batch normalization, and ReLU is used. This encoder re-encodes the original pyramid feature map $P_{i}$ to improve efficiency.

### 3.4. Decoder

**Figure 7:**
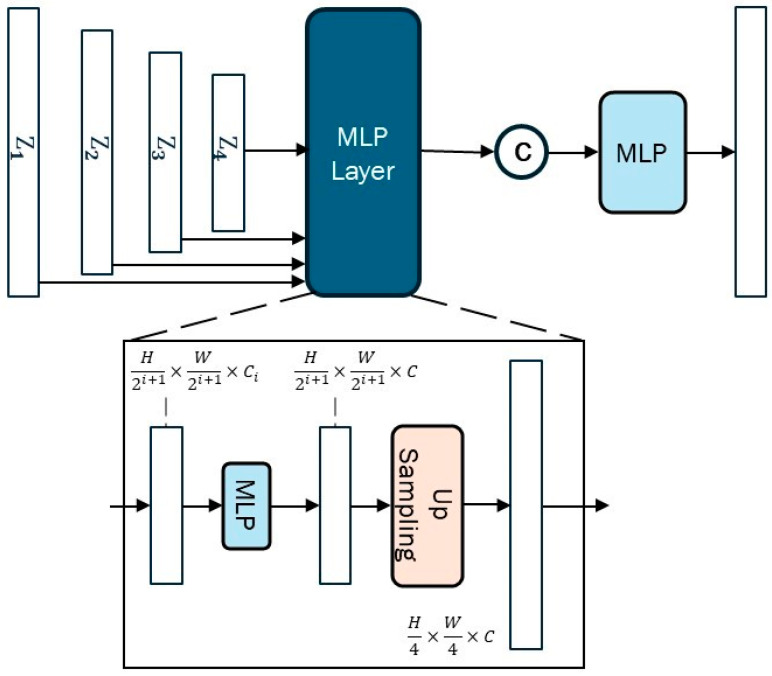
Decoder Architecture.

We designed a lightweight decoder composed solely of MLPs. Since the decoder receives multi-scale feature maps from the encoder, a unified dimensionality processing is required first. As shown in Figure 7, each scale of the feature map is initially processed through a 1 × 1 convolution layer to adjust the number of channels, ensuring that all feature maps have the same number of channels for fusion. Next, an MLP is used for feature fusion. The MLP consists of fully connected layers and ReLU. Each feature map $Z_{i}$ is then up-sampled. The up-sampled feature maps are aligned in spatial dimensions and channel dimensions. Four feature maps are concatenated along the channel dimension to form a high-dimensional fused feature map.

The fused feature maps are used as input to an MLP for further feature extraction and processing, producing the same number of channels as the number of classes. It is the prediction result of the semantic segmentation.

### 3.5. Loss Function

The imbalance between foreground and background proportions leads to overtraining background information during training. Hard background samples become valuable for optimization in the later stages of training, but their number is much smaller than that of easy samples. To ensure that the model focuses on hard samples in both foreground and background, we refine the Focal Loss [16] method using ${\left(1-p\right)}^{\gamma }$ as a weight to estimate hard samples. Here, $p\in \left[0,\;1\right]$ is the predicted probability, and γ is the modulating factor. For this pixel-level task with the foreground–background imbalance, our goal is to adjust the loss distribution without changing the total sum. To avoid the vanishing gradient, we dynamically normalized it. The formula is shown in Equation (9).

(9) $$\mathrm{Soft}\;\mathrm{Focal}\;\mathrm{loss}=\frac{1}{Z}\sum {\left(1-p_{i}\right)}^{\gamma }l\left(p_{i},y_{i}\right)$$

Here, $l\left(p_{i},y_{i}\right)$ denotes the cross entropy loss for the *i*-th pixel, calculated from the predicted probability $p_{i}$ and its true label $y_{i}$. The term Z represents the total number of pixels, and the loss weight for each pixel is given by ${\frac{1}{Z}\left(1-p_{i}\right)}^{\gamma }$. The Focal Loss differentiates between easy and hard samples, assigning a larger weight to the misclassified samples. When the predicted probability $p_{i}$ is high (indicating accurate predictions), the weight is smaller, preventing the model from overfitting on correctly classified samples, which are mostly background. When $p_{i}$ is low (indicating prediction error), the weight is higher.

Since the model makes many prediction errors during the initial training phase, it is difficult to determine whether these errors correspond to hard samples. It will lead to training instability and affect convergence performance. To address this issue, we introduce an annealing function for dynamic weighting. We propose three annealing function options, as listed in Table 2. From the experimental results exhibited in Section 6, we select the cosine annealing function. The loss calculation is shown in Equation (10).

(10) $$\overset{´}{l}\left(p_{i},y_{i}\right)=\frac{1}{Z}{\left(1-p_{i}\right)}^{\gamma }+A\left(t\right)\left(1-\frac{1}{Z}{\left(1-p_{i}\right)}^{\gamma }\right)\cdot l\left(p_{i},y_{i}\right)$$

Here, A(t) represents an annealing function related to the current training step *t* and $A(t)\in \left[0,\;1\right]$. When A(t) is 0, the loss function is determined solely by the original cross entropy loss $l\left(p_{i},y_{i}\right)$. When A(t) is 1, the loss function is determined by both the cross entropy loss and the weighting term.

The annealing step is a constant number that controls the annealing rate during the training process. The cosine function, a periodic function with a range of $\left[-1,\;1\right]$, is used here by dividing the time t by the annealing step and multiplying by π. This allows for a smooth transition of the cosine function from 1 to −1 as t changes. Adding 1 shifts the range to $\left[0,\;2\right]$, and multiplying by 0.5 scales it to $\left[0,\;1\right]$. The annealing function smoothly varies between $\left[0,\;1\right]$ as the training step t changes. As training progresses, A(t) smoothly decreases from 1 to 0, increasing the influence of the weights. This ensures that the model focuses on hard-to-learn samples only in the later stages of training, enhancing its stability and accuracy, improving overall performance, and reducing the risk of overfitting.

## 4. Dataset

We use a landslide remote sensing dataset provided by Sinotech Engineering Consultants, Inc., Taipei City, Taiwan, hereafter referred to as the LS dataset. The dataset was acquired by SPOT6 and has a spatial resolution of 2.5 m, making it a high-resolution remote sensing image. The dataset includes multispectral imagery, slope maps, and landslide annotations. It contains a total of 28 remote sensing images with sizes ranging from 8877×7637 to 23,043×20,771 pixels. The images were collected between 2004 and 2008 from ten watersheds in Taiwan, with landslide areas larger than 625 square meters. The watersheds include the Zhuoshui River, Hualien River, Beigang River, Daan River, Sichong River, Gaoping River, Taitung, Taipei City and New Taipei City, Dahan River, and Hsinchu. The goal is to detect landslide areas, with annotations provided as binary ground truth (0 for background, 1 for landslide areas), where the foreground accounts for only 0.029% of the pixels.

Due to the large image sizes of original images, which exceed hardware memory capabilities, the images were divided into smaller patches of 512 × 512 pixels with a stride of 256, resulting in a 50% overlap between patches. This produced a total of 43,174 remote-sensing image patches. To prevent overtraining on background information and to reduce training load, patches containing only background in the ground truth were removed. After removing background-only patches, the dataset was reduced to 13,612 remote-sensing image patches.

**Figure 8:**
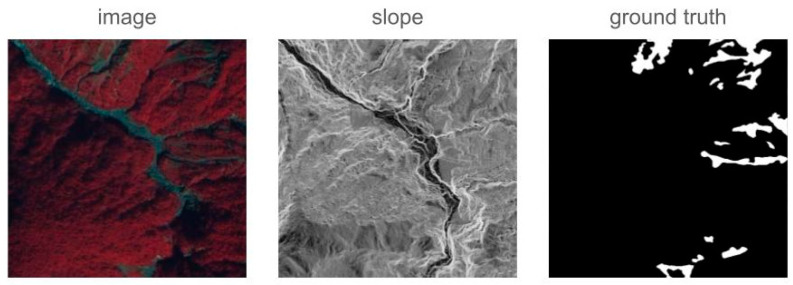
LS Dataset example (patch size: 512×512).

The slope map was normalized first and then concatenated with the original images for training to help the model distinguish between flat and sloped areas. Figure 8 shows the processed images. The original images are 8-bit TIF images with three bands, where the RGB channels correspond to the near-infrared, red, and green of the camera. The original slope maps are 8-bit grayscale images, and the normalized slope maps have their values distributed between 0 and 1. In the ground truth, white indicates landslide areas and black indicates the background.

## 5. Evaluation Metrics

To evaluate the performance of the model, the following commonly used validation metrics for image semantic segmentation were selected for analysis and comparison.

### 5.1. Precision Recall

*Precision* and *Recall* are crucial metrics for evaluating classification and semantic segmentation, especially in binary classification tasks. These metrics are calculated on a pixel-by-pixel basis. The calculation of *precision* and *recall* is listed in Equation (11) and Equation (12), respectively.

(11) $$Precision=\frac{TP}{TP+FP}$$

(12) $$Recall=\frac{TP}{TP+FN}$$

In Equation (11), True Positive (*TP*) means the model predicts 1, and the actual ground truth is also 1, which is a correct prediction. True Negative (*TN*) means the model predicts 0, and the actual ground truth is also 0, which is also a correct prediction. False Positive (*FP*) means the model predicts 1, but the actual ground truth is 0, which corresponds to an incorrect prediction. False Negative (*FN*) means the model predicts 0, but the actual ground truth is 1, which is an incorrect prediction.

### 5.2. IoU

IoU (Intersection of union) is not only used in object detection to determine the difference between the bounding box and the ground truth, but it can also be used to calculate the ratio of the intersection to the union of the predicted and ground truth areas on a pixel basis. A larger IoU value indicates more accurate predictions for that category. The formula is given in Equation (13).

(13) $$\mathrm{IoU}=\frac{Area\;of\;Overlap}{Area\;of\;Union}=\frac{TP}{TP+FP+FN}$$

IoU measures the overlap between model predictions and ground truth labels. It is simple and intuitive, and it is well suited for imbalanced data where some classes have very few pixels.

### 5.3. Pixel Accuracy

In semantic segmentation evaluation, Pixel Accuracy (PA) is a commonly used metric. It calculates the overall accuracy of the model across all pixels, as shown in Equation (14).

(14) $$\mathrm{PA}=\frac{\sum_{i=0}^{1}P_{ii}}{\sum_{i=0}^{1}\sum_{j=0}^{1}P_{ij}}$$

Here, $P_{ii}$ represents the total number of correctly predicted pixels. The terms $P_{ij}$, where i≠j, represents the model’s prediction errors. However, in remote sensing images, the number of background pixels exceeds that of the foreground significantly. The model’s performance in the majority class can mask its shortcomings in the minority classes, making PA a more unreliable metric.

### 5.4. F1 Score

In some cases, increasing *Precision* may decrease *Recall* and vice versa. When the model predicts fewer positive samples, *Precision* might increase, but *Recall* would decrease accordingly. The F1 Score combines these two metrics, providing a balanced evaluation that considers both *Precision* and *Recall*, as shown in Equation (15).

(15) $$F1\;\mathrm{score}=2\;\times \;\frac{Presicion\times Recall}{Presicion+Recall}$$

A higher F1 Score indicates better model performance, with the highest possible score being 1.

## 6. Results and Analysis

We used Python as the primary language and PyTorch as the deep learning framework. The training and evaluation were conducted using an NVIDIA GeForce RTX 2080ti GPU. Detailed hardware specifications and software versions are provided in Table 3 and Table 4.

In the experiments, all models were trained for 100 epochs using the Adam optimizer, with a weight decay of 0.01. The initial learning rate was set to 6 × 10^−5^, and the batch size was set to 2. A polynomial learning rate decay strategy was employed, with a patch size of 512 × 512. The number of channels in the FPN was set to 256. For the F–S relationship module, the dimension was set to 256. The default focusing factor γ in the focal optimization was set to 2, and the annealing step for the annealing function was set to 600 k. For data augmentation, during training, there was a 0.5 probability of random horizontal and vertical flips, image scaling, random cropping, and normalization of the RGB channels individually.

### 6.1. Results on the LS Dataset

We compared the proposed method with previous semantic segmentation research, using the metrics described in the previous section to evaluate model performance. Comprehensive experiments were conducted on the LS dataset, with results visually illustrated. Our model is compared with various CNN-based methods, multi-scale designed methods, and transformer-based methods. These methods range from classic to state-of-the-art techniques, including the classic FCN [17], Semantic FPN [30], Deeplab v3+ [31], PSPNet [13], DNLNet [32], EMANet [33], CCNet [34], DANet [35], UperNet [36], GCNet [37], the multi-scale designed HRNet [27], the SOTA method SegNeXt [15], the transformer-based Segformer [38], and FarSeg [9], which performs well in high-resolution remote sensing image scenes. The quantitative results listed in Table 5 demonstrate that our model significantly outperforms other methods across all five evaluation metrics on the LS dataset.

**Figure 9:**
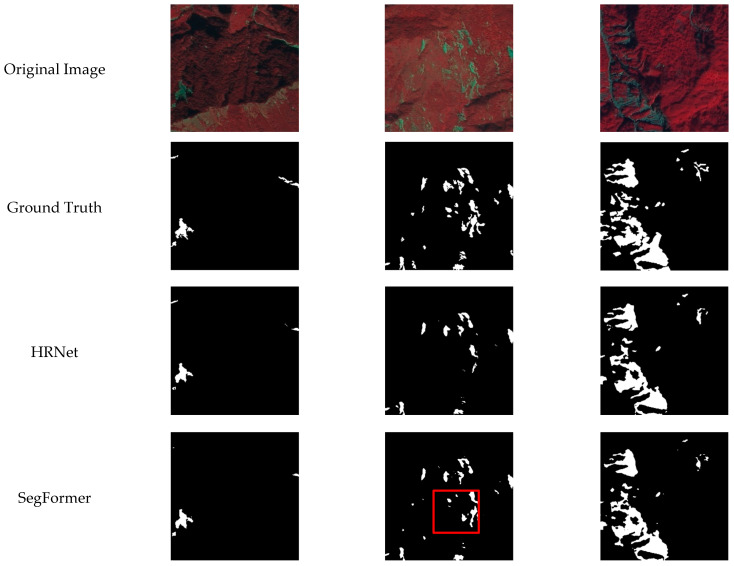

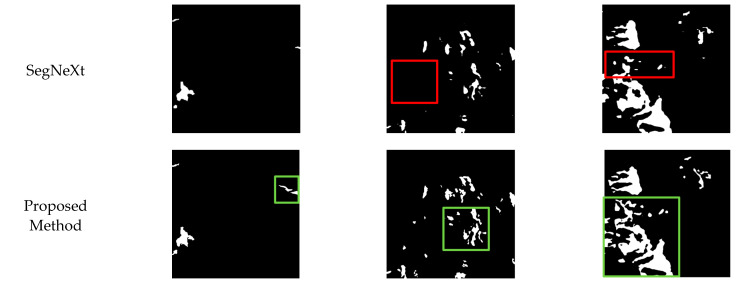
Visualization of Detection Results Using Different Methods. The detection results are highlighted using red boxes indicating the incorrect detections by other models and the green boxes indicating the better detection details of the proposed method.

Figure 9 shows a visual comparison between the proposed model and the top three models listed in Table 5. It is evident that our model performs better in detecting small objects and effectively predicts detailed areas. Other models frequently have false alarms and missed predictions. This is because our model establishes the relationship between the foreground and the scene, enabling the correct prediction of background areas similar to the foreground. Additionally, the multi-scale fusion design improves the model’s ability to predict small areas, providing finer segmentation of edges, even in complex background situations. In Figure 9, the green boxes highlight the details that our model can predict better, while the red boxes indicate the incorrect detections by other models.

**Figure 10:**
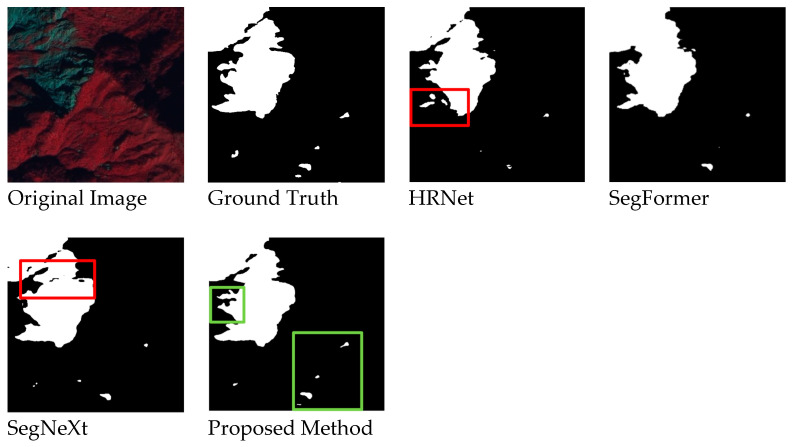
Detection Results for Large-Scale Objects.

Figure 10 shows a comparison when predicting large-scale objects. Other models often produce fragmented results (red boxes), while the detection results of the proposed method are more complete. Although SegFormer also achieves relatively complete detections for large-scale objects, our model performs better in handling details and small objects (green boxes). This indicates that our model can make more refined detections across different scales.

**Figure 11:**
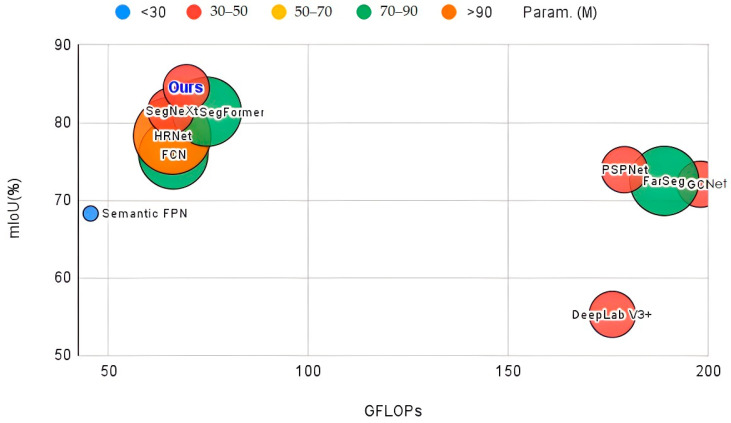
Comparison of Speed (FPS) and Accuracy (mIoU) on the LS Dataset.

Figure 11 illustrates the trade-off between speed and accuracy with an input image size of 512 × 512. Our model achieves a better balance between these two aspects. The radius of the circles in the figure represents the number of parameters, indicating that our model maintains high accuracy while achieving faster inference speed, showcasing high efficiency.

### 6.2. Ablation Study

This section discusses the contribution and effectiveness of each module proposed in this paper. The baseline model is SegNeXt, which uses the MSCAN as the backbone network and a Hamburger Decoder, a lightweight decoder, with Cross Entropy Loss for evaluation on the LS dataset. Unless otherwise specified, the same experimental settings are used.

#### 6.2.1. The Effect of Slope Maps

Table 6 shows the ablation experiments on the baseline method with and without the inclusion of slope data along with the remote sensing images. Due to the similarity in features between landslide and flatland areas with grass, soil, etc., incorporating slope maps to obtain elevation information is able to make landslide detection more accurate. The experimental results indicate that after adding the slope maps, four out of five metrics are improved. Although Recall decreased, the overall F1 Score increased, suggesting that slope maps play a positive role in enhancing the accuracy of landslide detection.

#### 6.2.2. The Effect of Different Modules

Table 7 presents the results of the ablation experiments for each module. An increase of IoU by 0.62% and F1 score by 0.45% can be observed after integrating the F–S Relation Module into the baseline method. Other evaluation metrics are also improved. It validates that the F–S Relation Module leverages scene features and contextual features to strengthen foreground features. Based on model (a), the proposed MLP decoder was added, further enhancing IoU by 0.26% and F1 score by 0.19%, while reducing 3.68 million parameters compared with model (b).

Model (d) incorporates focal optimization combining Focal Loss and annealing function. This achieved optimal performance, increasing IoU by 2.99% and F1 score by 2.13% compared to the baseline method, with little computation or memory overhead. This significantly alleviates the foreground–background imbalance issue in high spatial resolution remote sensing imagery. Moreover, it demonstrates the compatibility of focal optimization with the F–S Relation Module.

#### 6.2.3. Selection of Focal Optimization Methods

Table 8 compares different loss functions and annealing functions. Model (a) represents the baseline method using the cross-entropy loss function. Model (b) incorporates the weight ${\left(1-p_{i}\right)}^{\gamma }$ into the cross-entropy loss function to address the class imbalance issue, but the improvement is limited. This is because the Focal Loss function was originally designed for object detection, which is not ideal for pixel-level tasks like semantic segmentation. To adjust the loss distribution without changing the total loss and to avoid the vanishing gradient problem, normalization is applied, and annealing functions are introduced to help the model converge better. Method (c) normalizes the Focal Loss. Methods (d), (e), and (f) combine normalization with linear annealing, polynomial annealing, and cosine annealing functions, respectively. The results show that the cosine annealing function yields the best performance, significantly enhancing the overall model performance. This is because the cosine annealing function has a slow decay rate in the early and late stages of training, allowing for stable adjustment of the loss distribution, which aids in model convergence. This highlights the importance of adjusting the loss distribution and dynamically adjusting the loss function in mitigating the foreground–background imbalance issue.

#### 6.2.4. Comparison of Different Decoders

Table 9 compares different decoders. Method (a) is similar to FPN, where the four feature maps output from the pyramid and the F–S Relation Module are up-sampled to one-fourth the image size and then element-wise added. Method (b) uses the decoder from our baseline method. It only concatenates the last three layers and uses the Hamburger decoder to decompose the matrix, obtaining the prediction map through MLP. Compared to FPN, the baseline method shows slight improvements in all metrics. Method (c) improves upon the baseline method (b) by concatenating feature maps from all levels, resulting in small increases across all five evaluation metrics. Finally, Method (d) is the proposed decoder in this paper. Compared to other methods, it shows improvements in all performance metrics, indicating that the proposed enhancements are effective for image segmentation tasks.

### 6.3. Comparison with State of the Art Methods on Bijie Landlide Dataset

We compare the proposed model with existing state of the art methods using the Bijie landslide dataset, which is an open dataset. Table 10 shows the quantitative experimental results using the Bijie dataset. The statistics of MRFM [7] and ShapeFormer [8] are directly obtained from the cited references. The dash symbols in Table 10 represent that the statistics are not available from the original works. It can be observed that the proposed method exhibits superior performance on multiple indicators, especially on F1 score and mRecall.

## 7. Conclusions

This study addresses the challenges in semantic segmentation of high-resolution remote sensing images. General semantic segmentation often overlooks the imbalance between foreground and background and the issue of false alarm in remote sensing images. Therefore, we propose a semantic segmentation model designed specifically for high-resolution remote sensing images, applied to landslide detection. In the encoder part, a multi-scale convolutional attention mechanism is introduced, and an FPN framework is used to extract multi-scale features. To enhance the discriminability of foreground features and reduce false alarms, we introduced the Foreground–Scene Relation Module to model the relationship between foreground and scene. For the loss function, a weighting and annealing mechanism are introduced to improve the stability and accuracy of the model during training. The weighting mechanism emphasizes samples with larger prediction errors, making the model more focused on foreground samples and difficult samples in the background during training, thus alleviating the imbalance between foreground and background. The annealing mechanism dynamically adjusts the impact of the loss function as training progresses, enabling the model to learn more effectively. The overall design of the model architecture provides good detection results for objects of different sizes, especially in handling details. In quantitative experiments, our model achieved higher scores in Accuracy, IoU, F1, Precision, and Recall metrics, outperforming current state-of-the-art methods. Ablation experiments show that adding the above mentioned modules can indeed enhance the network performance, resulting in better detection results in all metrics.

## Figures and Tables

**Table 1:** The output feature maps dimensions, the number of channels, and the number of required blocks for each layer.

Stage	Output Size	$C_{i}$ (Output Channel)	*L* (# of MSCAN Block)
1	$\frac{H}{4}\times \frac{W}{4}\times C_{1}$	64	3
2	$\frac{H}{8}\times \frac{W}{8}\times C_{2}$	128	5
3	$\frac{H}{16}\times \frac{W}{16}\times C_{3}$	320	27
4	$\frac{H}{32}\times \frac{W}{32}\times C_{4}$	512	3

**Table 2:** Annealing function options.

Annealing Function	Formula	Parameter
Linear	$A\left(t\right)=1-\frac{t}{annealing\;step}$	annealing step
Poly	$A\left(t\right)={\left(1-\frac{t}{annealing\;step}\right)}^{decay\_factor}$	annealing step, decay factor
Cosine	$A\left(t\right)=0.5\times \left(1+\left(cos\frac{t}{annealing\;step}\pi \right)\right)$	annealing step

**Table 3:** Experimental Hardware and Operating System.

Hardware Environment	
CPU	Intel^®^ Core™ i9-9900K @ 3.60 GHz (Intel, Santa Clara, CA, USA)
GPU	NVIDIA GeForce RTX 2080ti (NVIDIA, Santa Clara, CA, USA)
RAM	64 GB
GPU memory	12 GB
OS	Ubuntu 18.04 LTS

**Table 4:** Software Environment Versions.

Software Environment	
Python	3.8.12
CUDA	11.3
Pytorch	1.11.0
Torchvisiony	0.12.0
Numpy	1.21.2
OpenCV	4.8.1
Pillow	9.0.1
MMCV	2.0.1
Docker	23.0.3

**Table 5:** Results on the LS dataset.

Model	PA (%)	mIoU (%)	mF1 (%)	mPrecision (%)	mRecall (%)
DeepLab V3+ [31]	97.69	55.31	60.86	74.88	57.19
DNLNet [32]	97.77	67.05	76.1	75.56	76.65
Semantic FPN [30]	97.92	68.29	77.36	77.2	77.53
EMANet [33]	97.94	68.79	77.88	77.31	78.47
CCNet [34]	98.07	70.03	79.09	78.75	79.44
DANet [35]	98.06	70.91	79.96	78.27	81.88
UperNet [36]	98.18	71.03	80.05	80.05	80.06
GCNet [37]	98.23	72.06	81.01	80.42	81.62
FarSeg [9]	98.45	72.49	81.37	84.84	78.56
PSPNet [13]	98.63	73.94	82.66	88.8	78.26
FCN [17]	98.61	75.9	84.37	85.39	83.42
HRNet [27]	98.78	78.33	86.35	87.08	85.66
SegFormer-B5 [38]	99.00	81.4	88.7	90.04	87.45
SegNeXt-L [15]	99.01	81.5	88.78	89.94	87.68
**Ours**	**99.18**	**84.49**	**90.91**	**91.24**	**90.58**

**Table 6:** Fusion of slope map ablation experimental results on the baseline.

Methods	PA (%)	mIoU (%)	mF1 (%)	mPrecision (%)	mRecall (%)
w/o slope	99.00	81.45	88.74	89.77	**87.76**
w/slope	**99.01**	**81.5**	**88.78**	**89.94**	87.68

**Table 7:** Results of the Ablation Experiments for Different Modules.

Methods	F–S Relation	Ham. Decoder	MLP Decoder	Focal Opt.	PA (%)	mIoU(%)	mF1 (%)	mPrecision (%)	mRecall (%)	Params. (M)
(a) Baseline	-	✓	-	-	99.01	81.5	88.78	89.94	87.68	**48.8**
(b) w/F–S Relation	✓	✓			99.04	82.12	89.23	90.27	88.24	52.78
(c) w/F–S Relation and MLP Decoder	✓		✓		99.06	82.38	89.42	90.31	88.57	49.1
(d) w/F–S Relation, MLP Decoder and Focal Opt. (Proposed)	✓		✓	✓	**99.18**	**84.49**	**90.91**	**91.24**	**90.58**	49.1

**Table 8:** Ablation Study of Different Loss Functions and Annealing Functions.

Methods	PA (%)	mIoU (%)	mF1 (%)	mPrecision (%)	mRecall (%)
(a) w/o Opt. (Cross Entropy Loss)	99.00	81.45	88.74	89.77	87.76
(b) Focal Loss	99.03	82.01	89.15	89.74	88.58
(c) +Norm.	99.04	82.15	89.25	89.94	88.58
(d) +Norm. + Linear Annealing	99.02	81.96	89.11	89.69	88.55
(e) +Norm. + Poly Annealing	99.04	82.19	89.28	90.22	88.39
(f) +Norm. + Cosine Annealing	**99.18**	**84.49**	**90.91**	**91.24**	**90.58**

**Table 9:** Comparison of Different Decoder Experiment Results.

Decoder	PA (%)	mIoU (%)	mF1 (%)	mPrecision (%)	mRecall (%)
(a) FPN	99.03	82.05	89.18	89.94	88.44
(b) Hamburger (baseline)	99.04	82.12	89.23	90.27	88.24
(c) Hamburger v2	99.04	82.23	89.31	90.07	**88.57**
(d) Proposed	**99.06**	**82.38**	**89.42**	**90.31**	**88.57**

**Table 10:** Quantitative Results using Bijie Dataset.

Methods	PA (%)	mIoU (%)	mF1 (%)	mPrecision (%)	mRecall (%)
MRFM [7]	-	**87.47**	87.98	**90.55**	85.56
ShapeFormer [8]	-	78.72	88.11	86.74	89.52
PSPNet [13]	97.54	78.18	86.43	86.52	86.33
FCN [17]	97.23	76.94	85.47	83.81	87.35
Ours	**97.75**	80.52	**88.20**	86.53	**90.10**

## Data Availability

The data presented in this study are available on request from the corresponding author. The data are not publicly available due to privacy restrictions.
